# Notes and News

**Published:** 1896-04-25

**Authors:** 


### Notes and News.

(Collected and Communicated.)

### HOSPITAL MEETINGS.

The Annual Dinner of the Royal General Theatrical Fund will take place at the Hotel Metropole on May 28th under the presidency of Lord Russell of Killowen.

A Festival Dinner in aid of the funds of the Royal Albert Orphan Asylum, Bagshot, will be held at the Savoy Hotel, on Thursday, May 21st, at 7.30 p.m., under the presidency of H.R.H. the Duke of York.

The Annual Festival Dinner in aid of the funds of the Royal Hospital for Incurables, Putney Heath, will be held at the Whitehall Rooms, Hotel Metropole, on May 19th, Mr. H. J. Allcroft in the chair.

A Festival Dinner in aid of the East London Hospital for Children, Shadwell, E., will be held at the Hotel Cecil, Strand, W.C., on Wednesday, May 13th, at 7.30 p.m., Mr. John Aird, M.P., in the chair.

The Queen has signified her intention of giving one hundred guineas to the re-endowment fund of Guy's Hospital.

Owing to the outbreak of small-pox at Alford, vigorous steps are being taken to establish a cottage hospital for the reception of patients.

A spacious infirmary has just been added to the Walsall Workhouse. It will accommodate 120 patients, and the cost of the building, including furnishing, was £8,000.

A severe epidemic of enteric fever has broken out at Allahabad. Suspicion is cast upon the vessels used for the storage of the water, the supply itself proving satisfactory after examination.

The French Academy of Medicine have decided to divide the prize for the discovery of a remedy for diphtheria between Dr. Roux and Dr. Behring. The prize, which amounts to 250,000 francs, was offered by Mons. and Mme. Victor St. Paul.

The chairman of the Council of Management of the Royal Chest Hospital, City Road, E.C., has sent a donation of 300 guineas towards the fund being raised in connection with the festival dinner, to be held at the Hotel Metropole on May 26th.

It has been questioned whether the fighting force for the Soudan is equal to the probable demands upon it, but no doubt is expressed as to the provision for the sick and wounded. The seven military hospitals of the Egyptian Army are well and fully equipped, and thorough preparations are being made to provide hospitals along the line of advance. Some hospitals will be fixed, and others moveable. Eventually the transport of the sick will be by boat, but until this method is organised camels will be utilised.

Cholera has again broken out at Alexandria.

The Fishmongers' Company have made a grant of £105 to the Royal Albert Orphan Asylum, Bagshot.

The new temporary wing of the Miller Hospital was opened last Saturday in the presence of a large assemblage. This building will be used for casualties until the establishment of the permanent erection, for which funds are not yet forthcoming.

Sir Henry Hawkins and Professor Atkinson are organising a scheme to provide a hospital for animals. The building will be dedicated to the memory of Jack, "the judge's dog." The proposal will meet with much sympathy, as a home where skill, care, and kindness can be secured for pets has long been wanted.

The London Buisson Institution is now opened at Upper Norwood. It is established to promote the "sweating bath" treatment of hydrophobia as opposed to Pasteur and other methods. Patients are treated without fees, no in-patients being received, except under special circumstances.

After the luncheon to be given by the Lord Mayor at the Mansion House on Monday, June 8th, to the Prince of Wales, the Duke of York (the Master), and the other Elder Brethren of the Corporation of the Trinity House, the Prince of Wales, who is Vice-Patron of the Metropolitan Hospital Sunday Fund, will, on behalf of the council, present an address and album to Mr. Henry C. Burdett, in appreciation of the interest he has taken in the Fund for a long period, and especially of his strenuous exertions and personal influence last year in obtaining subscriptions, raising the Fund to the unprecedented sum of over £60,000.

Mr. Arthur H. Shaw, the hon. secretary of the Memorial Cottage Hospital, Leek, Staffordshire, has set an example which, if generally followed, must prove beneficial to cottage hospitals as a class. In the twenty-second annual report he has compared the expenditure of the Leek Hospital with that of other similar institutions as given in the new edition of "Cottage Hospitals," published by the Scientific Press, and has given its title-page in a footnote, so that all his subscribers may know where to obtain it and the cost. As this book is the only one published on the subject, which it treats exhaustively, Mr. Shaw's action is likely to prove popular with his supporters and helpful to the Leek Cottage Hospital.

It is impossible to express an opinion regarding the deadlock which has occurred at the Adelaide Hospital, South Australia, until more details come to hand. It would appear from telegrams which have been received that the honorary medical staff have resigned in a body, that they are supported in their course by the profession in the place, and that the Government are proposing to import paid men to do their work at the hospital. We fear that this means some attempted intermeddling on the part of the Government in the internal management of the hospital—a not infrequent cause of difficulty in the colonies, where hospitals are often Government institutions, only partly supported by subscription, and, although managed by local committees, only so managed under constant liability to interference from headquarters.

